# Worked examples of alternative methods for the synthesis of qualitative and quantitative research in systematic reviews

**DOI:** 10.1186/1471-2288-7-4

**Published:** 2007-01-15

**Authors:** Patricia J Lucas, Janis Baird, Lisa Arai, Catherine Law, Helen M Roberts

**Affiliations:** 1 School for Policy Studies, University of Bristol, 8 Priory Rd, Bristol BS8 1TZ, UK; 2 MRC Environmental Resource Centre, University of Southampton, UK; 3 Child Health Research & Policy Unit, City University, UK; 4 Institute of Child Health, University College London, UK

## Abstract

**Background:**

The inclusion of qualitative studies in systematic reviews poses methodological challenges. This paper presents worked examples of two methods of data synthesis (textual narrative and thematic), used in relation to one review, with the aim of enabling researchers to consider the strength of different approaches.

**Methods:**

A systematic review of lay perspectives of infant size and growth was conducted, locating 19 studies (including both qualitative and quantitative). The data extracted from these were synthesised using both a textual narrative and a thematic synthesis.

**Results:**

The processes of both methods are presented, showing a stepwise progression to the final synthesis. Both methods led us to similar conclusions about lay views toward infant size and growth. Differences between methods lie in the way they dealt with study quality and heterogeneity.

**Conclusion:**

On the basis of the work reported here, we consider textual narrative and thematic synthesis have strengths and weaknesses in relation to different research questions. Thematic synthesis holds most potential for hypothesis generation, but may obscure heterogeneity and quality appraisal. Textual narrative synthesis is better able to describe the scope of existing research and account for the strength of evidence, but is less good at identifying commonality.

## Background

The inclusion of qualitative data in systematic reviews is an area of ongoing methodological development [1-3], with particular problems arising for reviews attempting to synthesise quantitative with qualitative data. The Cochrane qualitative methods group [2] suggests four areas in which development is needed; (1) searching, (2) critical appraisal, (3) synthesis/summary, and (4) loss of research context. This paper aims to contribute to development in the synthesis of qualitative and quantitative data. Alternative models and vocabularies of synthesis are emerging [3-9], but standard methods for combining different data types from the qualitative and quantitative research traditions have not yet been agreed [8].

Innovative methods are often developed during the course of research, but in general, papers report methods only briefly. As a result, the material that could inform learning is more often to be found in filing cabinets than in journals. In this paper we aim to distinguish between "the trivial and non-trivial points of divergence" p.31 [4] by providing worked examples of two methods of evidence synthesis (thematic and textual narrative) tested in one systematic review.

## Methods

A systematic review of lay views about infant size and growth was undertaken as part of a series of interlinked reviews examining the evidence for associations between early growth and a number of later outcomes. The systematic review of views included both qualitative and quantitative studies.

Study methods and findings are reported in greater detail elsewhere [10-13]. Standard systematic review methods were employed, following guidance from the Centre for Reviews and Dissemination [14] and from an advisory group with backgrounds in public health, paediatrics, infant nutrition, qualitative and quantitative methods, systematic reviewing, and including representatives from user groups. Twelve databases were searched using terms for growth, height, weight and infancy as well as appropriate methodological terms. 2,694 abstracts were retrieved, from which 19 studies met the inclusion criteria for the review.

Two researchers independently extracted findings by interrogating each study using the following questions developed from the aims of the review:

1. What is healthy growth/size?

2. How important is growth/size to participants?

3. What concepts are used to define healthy growth/size?

4. How do participants assess growth/size?

5. Where does growth lie among priorities for child health?

6. What information influences views/behaviour?

7. Who influences views/behaviour?

Directly reported participant data (e.g. verbatim quotations or scores on attitudinal scales) and author interpretations were recorded separately, to retain the richness or 'thickness' of the contributing data. 'Thickness' in this context refers to the kinds of relatively detailed descriptions and contextual material which help the reader to make judgements about the trustworthiness of the data, particularly when applying it to different contexts [15,16]. Study characteristics and quality assessment were summarised (for examples see Table 3). There is vigorous debate on whether qualitative research can be assessed using standard quality criteria, or whether this process is contrary to the nature of qualitative enquiry [17]. While the controversy on the use of critical appraisal in systematic reviews including qualitative data lies beyond the scope of this article, with views ranging from those who believe that critical appraisal is core to qualitative synthesis [18] to those who, like Barbour [19] consider that critical appraisal of qualitative research can be reductionist, it is notable that there is general agreement that a checklist approach to critical appraisal can bring its own problems, particularly in relation to transparency in assessing interpretative work. We took the view that applying quality criteria rigidly would be likely to exclude relevant studies that had failed to comply with a particular reporting regime. Thus, all studies meeting our inclusion criteria listed were included and quality appraisal was used at the data synthesis stage contributing to strength of evidence.

**Table 3:** Example study summaries

Study	Participants Location Design	Aims (where possible verbatim) Appraisal of Methods	Infant age Participation rate Attributable quotations	Setting Sampling Triangulation	Key findings
Baughcum et al. 1998	16 dieticians, 6 WIC* mothers, 8 teenage WIC mothers Kentucky, USA Qualitative (focus groups)	"to identify maternal beliefs and practices about child feeding that are associated with the development of childhood obesity" Design allowed for exploration of subjective experience.	12–36 months age Not stated for mothers, 95% for dieticians Not attributable to individuals	WIC* clinic and WIC* nurses Risk of bias as sampling restricted to health clinic users Not stated	Mothers were not concerned about overweight in their children. This was perceived as a problem by dieticians and study authors.
Baughcum et al. 2001	454 mothers, 258 attending WIC* and 196 attending private child health clinics. Cincinatti & Kentucky, USA Quantitative attitudinal (closed questionnaire)	"to determine if the factor scores [from questionnaire under development] were associated cross-sectionally with (1) the child being overweight at the time of the survey (2) maternal obesity, and (3) lower socio-economic status." Design did not allow for subjective views.	11–24 months, but considering retrospectively to first year. 98% Not attributable to individuals	Health clinics (WIC* or private) Risk of bias as sampling restricted to health clinic users Not stated	Mothers were more concerned about under eating and underweight, although where children were overweight there was concern about overeating and overweight.

* Special Supplemental Nutrition Program for Women, Infants and Children

Two methods were proposed for synthesis of findings, textual narrative and thematic, both of which the advisory group agreed were appropriate to our needs. The first, the textual narrative approach, involves a commentary reporting on study characteristics, context, quality, and findings, using the scope, differences and similarities among studies were used to draw conclusions across the studies, whilst the second, the thematic approach, groups data into the themes. Given the relatively small number of studies located, it was feasible to test both methods. Findings from the review are provided briefly for illustration, but the focus of this paper is on the process of synthesis and a comparison of methods used. The two reviews ran in tandem, as the thematic review needed time for response and comparison between reviewers.

## Results

### Worked Example 1 – Textual Narrative Synthesis

Factors identified by the research team from the research literature as likely to affect views on infant growth were used to define a number of sub-groups. These were:

1. Relationship between participant and infant (e.g. mothers, other family members, health professionals, unrelated others)

2. Weight status of participant

3. Ethnicity of participant

4. Age of infant

5. Views about infants considered 'high risk' at birth i.e. those born too small or too early, or who were placed in a neonatal intensive care unit (NICU)

6. Weight/growth status of infant after birth

7. Mode of infant feeding (breast fed, bottle fed, weaned)

Using agreed versions of quality appraisal and extracted data a textual narrative synthesis was undertaken by a single researcher (PL). Each study within a sub-group was described in a commentary reporting on study characteristics, context, quality, and findings. The scope, differences and similarities among studies were used to draw conclusions across the studies (the synthesis). Drawing conclusions across studies was not always possible due to study heterogeneity and lack of data. A worked example of the process is shown in Table 1.

**Table 1:** Stepwise textual narrative synthesis

**Step 1**: Study grouping. Studies belonging to each of the sub-groups were identified. For example studies classified by relationship between participant and infant were: a) Mothers;16 studies [20,28–41] b) other family members;1 study [20] c) health professional; 2 studies [28,42] d) unrelated others; 2 studies [43–45]
There was overlap between sub-groups. For example a study of mothers 2 months after their infants were admitted to NICU would fall within 3 groups, determined by the 'participants being mothers', the 'age of the infant's and the fact that the infants were considered 'high risk'.
**Step 2**: Study commentaries produced. These commentaries summarised key aspects of the studies in relation to the sub-group within which they were included. For example a study of mothers' views:
A study by Baughcum and colleagues[28] reported on focus groups conducted with 14 mothers attending WIC clinics (Special Supplemental Nutrition Program for Women, Infants and Children) in USA with infants aged 12–36 months. The study focussed on maternal attitudes to feeding and proposed an association between these and overweight in their babies. The study design was judged adequate, although the bias introduced by sampling from WIC clinics was not discussed by study authors. Authors concluded that mothers are more concerned about under- than overweight; two supporting quotations stated that weight gain is always good, because it means children are eating.
**Step 3**: Sub-group synthesis produced. For example the views of mothers:
Most of the studies in this review (16/19) explored the views of mothers. The mothers, varied in terms of the age of their infants, the present and past health status of their children, their country of residence, their country of origin, income level, socio-economic status (SES), and number of children. North American Caucasians made up most of the sample. Background data for participants was often unknown, unreported or incomplete. Sampling strategies in the studies created difficulties in interpreting findings. For example, three studies explicitly set out to sample low income groups[28,29,37] using WIC clinics to achieve this. To be recruited to these studies, families needed to have a low income, but also needed to register for the WIC programme and attend clinics. This strategy is likely to selectively recruit participants[29]. Studies typically did not allow comparison between groups (for example those from different ethnic backgrounds) because findings were not reported separately.
Growth and size were concerns for mothers, particularly achieving average or normal growth. Mothers used a variety of sources of information to define norms, including growth charts, clothing and familial patterns of growth/size. There was evidence of concern for underweight, but the extent of concern about overweight was unclear.

### Findings – Textual Narrative Synthesis

We noted that unrelated members of the public tended to prefer infants of mid-range body sizes, but the evidence to support this observation was thin. Families of children with poor growth were acutely aware of growth as a problem; they monitored growth and discussed it with others. They desired "normal" growth in their child, and looked for ways that they could interpret the infant's growth as normal (for example finding members of the extended family who were of similar body shape). The most common method of assessing size in all sub-groups was by comparison with others, although the use of growth charts and physical measurement were also important for those with children with poor growth including babies born too small or too early. However, growth and size in themselves were low among concerns about such 'high risk' babies. The predominance of those with 'high risk' infants may explain our conclusion that growth was low among priorities for mothers of younger infants (aged 0–3 and 3–6 months). Among older children (more than 12 months) with poor growth there was concern among parents. Parents wanted to see good growth in their children, but they also considered love, attention, good health and good diet as important.

We judged that we had insufficient data to draw conclusions about the views of family members other than mothers, health professionals, or to compare the views of participants of different weight, ethnicity, or toward breast versus bottle fed infants.

### Worked Example 2 – Thematic Synthesis

Thematic synthesis was undertaken by two researchers, LA and PL. Findings from all studies were collated under the 7 questions used in data extraction. Each researcher independently conducted a thematic analysis using these findings. On initial discussion of themes, researchers judged that there was repetition between the data extraction questions, and that data referred to four broad areas of enquiry:

1. Understanding healthy growth/size

2. Assessment of growth/size

3. Concerns about growth/size

4. Influences on views, behaviour, interpretations of growth/size

Data and themes were grouped into these areas and emerging themes were then considered for relevance, presence across studies, 'thickness' and duplication. This process was repeated until researchers were satisfied that all data could be interpreted within these themes and an agreed version reached. A worked example of the process is shown in Table 2.

**Table 2:** Stepwise thematic synthesis

**Step 1**: Data collated under question derived from study aims and independently reviewed by researchers, for example observations concerning 'understanding healthy growth/size' included:
-the word 'normal' frequently used by mothers e.g. "you don't want him to be seven feet tall, you just want him to be normal, like everyone else." [20] -mothers of children with faltering growth were reported as tending to underestimate the extent of their child's thinness -a preference for mid-range body sizes
**Step 2**: Themes produced by each researcher were compared and a consolidated list produced. For example, themes under 'understanding healthy growth/size' included:
Normal for family; *"you look at me and his father, so he's not gonna be little either." *(low income mother)[28] Predeteremined: *"he's finally taking the form he's supposed to have." *(WIC mothers)[37] Normal for population;*" normal, like everyone else." *[20] Preference for mid-range body sizes[43] Reaching "normal" size and development was key for many parents particularly for parents of low birth-weight infants
**Step 3**: Clustering of themes. When the themes falling under each review question were clustered around common dimensions. For example clusters under 'understanding healthy growth/size' were:
Themes referring to norms of healthy size or growth Themes which explained differences from these norms (e.g. 'medical' causes) Themes which referred to seen (e.g. nutrition) or unseen (e.g. hereditary characteristics) determinants of size/growth
**Step 4**: Agreed synthesis produced; example assessment of size/growth
*Constructing size norms*
Seven studies reported data on how participants assessed or defined normal size [20,28,31,37,38,42–44] Four themes emerged;
1. Medical definitions, including the use of growth charts [37,38,42] *"I take her to clinic where they measure her height and her weight. They show me ... what is the normal height for children her age" (*WIC mother) 2. Comparisons to other children in the community. [20,31,43,44] *"you just want him to be normal, like everyone else." (mother) *[20] 3. Comparison with family members. [28,37] *"She's just a little below average as far as the children in the family" (*WIC mother) [37] 4. One study reported use of clothing sizes; *"if they are not fitting in the clothes they should be fitting in, they're not average" (*WIC mother) [37]

### Findings – Thematic Synthesis

Across the thematic synthesis the predominant concern of participants was normality. This was seen through the creation of norms of growth and models to explain difference. This was conducted across physical, observable characteristics, but included physical unobservable (such as underlying health status) and non physical (such as emotional care) dimensions. Where growth differed from the norm and a plausible explanation could not be found, for example among families of those with faltering growth [20], growth became an important concern for parents.

Data from across studies could be usefully combined in this method, for example in listing all the sources of influence on behaviour or views found. Family, other parents and friends, information from the infant themselves, health professionals, clothing sizes, magazines, books, radio, TV and their religious beliefs were all important to some, but the relative importance of these could not be explored.

### Strengths and limitations of our study

While the data extraction and thematic synthesis was undertaken by two researchers working independently, only one of these researchers (employed to work on the qualitative aspect of the review) worked on the narrative synthesis with a second researcher discussing the work as it progressed. Whether the findings might be different with more than one researcher working on both syntheses, or researchers not involved in the data extraction doing the syntheses, or the syntheses being carried out in a different order, are themselves research-able (if rather expensive) questions, as is the issue of whether the immersion of one researcher in the data at every stage a strength (as we believe it to be) or a source of bias.

## Discussion

Reassuringly, the conclusions to which these analyses led us about lay perspectives were largely similar across the thematic and textual narrative synthesis. Whether using a different research team, or a larger number of reviewers, would have produced different results is itself a researchable question. However, in this case conclusions from both analyses were dominated by importance of having babies that were a 'normal' size, leading to interest in monitoring of growth in a number of ways and, sometimes, to concern that there was an underlying problem leading to 'abnormal' growth. While the general conclusions were the same, the process and the implications of the two types of synthesis differed.

### Strengths and Weaknesses of Textual Narrative Synthesis Methods

A textual narrative approach typically groups studies into more homogenous groups. This technique has been particularly successful in synthesising different types of research evidence (e.g. qualitative, quantitative, economic). Examples include a number of reviews carried out by the Evidence for Policy and Practice Information and Co-ordinating Centre (EPPI-Centre) [21-23], reviews of tobacco use and exposure to tobacco smoke [24], reviews of ultrasound in pregnancy [25] and of communication between health care professionals and patients about prescribing [26].

In our review, the textual synthesis proved a useful way to describe difference in the included studies, making explicit the diversity in study designs and contexts. The textual narrative review also described gaps in the literature, both by showing where evidence was absent and by making an evaluation of the strength of evidence in different areas. Using this method enabled us to comment on, for example, the ethnic uniformity of participants, and the lack of evidence collected regarding mode of feeding.

However, transparency remained a problem. For example, decisions about which sub-groups to use for synthesis of individual studies rely on judgements, albeit ones which can be informed by the scientific literature and by lay views. While we sought to make the decision making process clear, interpretation and judgement, which are not fully susceptible to external scrutiny, lie at the heart of the process.

### Strengths and Weaknesses of Thematic Synthesis

The strengths of the thematic synthesis lie in its potential to draw conclusions based on common elements across otherwise heterogeneous studies. This synthesis is potentially more accessible for the reader than a textual synthesis. Conclusions from this thematic synthesis fulfil an important research aim of qualitative research in generating hypotheses, an area to which traditional systematic reviews are poorly suited [27].

However, pooling findings in the thematic synthesis risks masking the shortcomings of the individual studies that make up the review. Although descriptions of study characteristics and quality appraisal were presented alongside synthesised findings, the synthesis process obscured these in the conclusions. We believe that further debate about the reliability of this approach would be useful. On the one hand, the hypotheses that emerge from this synthesis draw on a broader body of views than any single study (as in a meta-analysis) and may therefore increase reliability; on the other, we risk making strong conclusions based on a group of studies none of which is in itself reliable on the grounds of quality or diversity of context. This method may also be poor at examining contradictions, as well as commonalities, in the data and at highlighting gaps in the evidence.

## Conclusion

The selection of synthesis method for systematic reviews such as this may depend on the aims of the synthesis. For the purpose of generating future research hypotheses, the thematic synthesis appears to hold the greatest potential; describing common themes and providing a possible structure for new research. In contrast, the textual narrative synthesis might be better suited to reviews which aim to describe the existing body of literature; identifying the scope of what has been studied, the strength of evidence available, and gaps that need to be filled.

## Competing interests

The authors declare no competing interests.

## Authors' contributions

CL, JB, HR, obtained funding for the study. All authors were responsible for the concept and design of the study. PL, and HR carried out the review work with assistance from all other authors. PL, LA & HR were responsible for the interpretation of findings. PL and HR produced the first and subsequent drafts of the paper, all authors were responsible for critical revision of the manuscript.

## Pre-publication history

The pre-publication history for this paper can be accessed here:


